# SeqSVM: A Sequence-Based Support Vector Machine Method for Identifying Antioxidant Proteins

**DOI:** 10.3390/ijms19061773

**Published:** 2018-06-15

**Authors:** Lei Xu, Guangmin Liang, Shuhua Shi, Changrui Liao

**Affiliations:** 1 School of Electronic and Communication Engineering, Shenzhen Polytechnic, Shenzhen 518060, China; csleixu@szpt.edu.cn (L.X.); sshua@szpt.edu.cn (S.S.); 2 Key Laboratory of Optoelectronic Devices and Systems of Ministry of Education and Guangdong Province, College of Optoelectronic Engineering, Shenzhen University, Shenzhen 518060, China; cliao@szu.edu.cn

**Keywords:** antioxidant protein, primary sequence, support vector machine, maximum relevance maximum distance, feature selection

## Abstract

Antioxidant proteins can be beneficial in disease prevention. More attention has been paid to the functionality of antioxidant proteins. Therefore, identifying antioxidant proteins is important for the study. In our work, we propose a computational method, called SeqSVM, for predicting antioxidant proteins based on their primary sequence features. The features are removed to reduce the redundancy by max relevance max distance method. Finally, the antioxidant proteins are identified by support vector machine (SVM). The experimental results demonstrated that our method performs better than existing methods, with the overall accuracy of 89.46%. Although a proposed computational method can attain an encouraging classification result, the experimental results are verified based on the biochemical approaches, such as wet biochemistry and molecular biology techniques.

## 1. Introduction

Permeability is an intrinsic nature of a normal cell membrane. Not only the water and oxygen are allowed to flow into the cell freely, but also the carbon dioxide and other waste products (uric acid, water, and etc.) can pass through the cell membrane. The free radicals exist in metabolic process, X-rays, air pollutants, cigarette smoking, etc. [1]. They are unstable before they find atoms for neutralization. Since the skin is damaged outside every day, the free radicals are harmful to the cells of the skin. They can create a chain with the beginning of oxidative damage, and then the cells are destroyed. 

Antioxidant proteins can neutralize free radicals to make them stable. Research shows that antioxidant proteins play an important role in terminating cellular and DNA damage caused by free radicals [2]. The damage caused by free radicals is the source of aging and various diseases [3,4,5]. Thus, research on antioxidant proteins has been paid more attention recently.

Although some micronutrients (vitamins) have been recognized as antioxidant molecules, such as vitamin E, vitamin C, etc., it is still necessary to identify effective proteins with antioxidative characteristics. Unfortunately, it is time-consuming to predict the antioxidant proteins by biochemical experiments. The computational method for prediction has been paid more attention recently, such as SNPdryad, used for predicting deleterious non-synonymous human SNPs (Single Nucleotide Polymorphisms) [6,7]. The computational methods used for identifying antioxidant proteins are expected, especially for the cases with large amount of protein sequence data. A method based on star graph topological indices was proposed to handle the problem [4], and the results are encouraging. However, the sequences in [4] are reused in the experiments, which the results are likely to be overestimated. Furthermore, a naive Bayes model was proposed by Feng et al. [8] to predict antioxidant proteins. The model proposed in [8] is based on optimal dipeptides, and the accuracy is 66.88% evaluated by jackknife test. The accuracy of AodPred [9], based on g-gap dipeptide composition, is 74.79%. As we have known, the accuracy of antioxidant proteins can be improved. 

The experimental results of previous work show that the performance of predicting antioxidant is related to the representation of proteins and the classifiers. The sequence information of proteins should be described precisely in the process of protein representation [10,11]. The study of protein representation has been paid more attention these years, such as the amino acid composition (AAC) model used in [12,13,14], g-gap dipeptide composition, proposed in [15], 400D [16], 188D [17], and others. The protein is represented by a simple vector in AAC model, whose elements represent the normalized occurrence frequency of the native amino acid in the peptide chain. As a result, the sequence information is lost in the AAC model. G-gap dipeptide composition [18] is a sequence-based feature extraction method for protein representation, which has been used widely in the realm of bioinformatics [2,9,17,19,20,21,22,23,24]. 400D is a method that represents the occurrence frequency of two consecutive amino acids, which is used in [16] to identify anticancer peptides. 188D [17] contains 188 features, including the physicochemical property attributes, the occurrence frequency of amino acid information, and others. The features can be combined together for keeping more information, as in [25,26]. In our work, the protein is described based on the physicochemical properties [17], and there are, totally, 188 dimensions used for protein representation. However, the results of experiments show that there may be redundancy between the features, so it is necessary to reduce the dimensionality of the features [27]. The redundancy is also considered in our work by maximizing the relevance and the distance between the features [28], and number of features is reduced to 132. The problem of imbalance class is considered in our work, and the dataset is processed by SMOTE (synthetic minority oversampling technique) method. The experimental results demonstrate that the reduced features attain higher accuracy than 188D. In other words, the accuracy of SeqSVM using 188D is 88.68%, while the accuracy of SeqSVM is improved to 89.46% using the method of MRMD (maximum relevance maximum distance) to select the features. Compared with AodPred [9], the accuracy of our method is better than that of AodPred, whose accuracy is 74.79%.

Above all, the contributions of our work include as follows:

(1) A computational method called (SeqSVM) is proposed to predict antioxidant proteins, which is based on the primary sequence features proposed in [17]. The features are described by the physicochemical properties and sequence information of the protein, the dimensionality of the extracted features is 188, so the feature used here is called 188D.
(2) There is redundancy in the 188D feature. In the manuscript, the features are selected by maximum relevance maximum distance method [28]. The features will be kept which can maximize the Pearson’s correlation coefficient and the distance between attributes. The experimental results show that the performance of the method using selected features is competitive, or even better than that of the method using 188D.
(3) The proposed method uses support vector machine for antioxidant protein prediction. The experiments demonstrated that our proposed method performs better than existing methods with the accuracy of 89.46%. The best result of existing work is 74.79% proposed by Lin et al. [9].

The rest of the paper is organized as follows. The experimental results are discussed and analyzed in Section 2. Section 3 introduces the dataset used in the proposed work, the classification method, SMOTE processing, sequence representation, and performance evaluation. Finally, a conclusion is given in Section 4.

## 2. Results and Discussion

### 2.1. Comparison with Existing Methods

Our proposed method (SeqSVM) is compared with existing methods. Table 1 shows the comparison of our method with the existing method, on accuracy. The dataset is processed by SMOTE method to make a balance between the antioxidant samples and non-antioxidant samples in SeqSVM. For the purpose of removing the feature redundancy, the features are selected by max relevance max distance principle. In Table 1, the accuracy of our method with SMOTE processing and MRMD is 89.46%. Naive Bayes method is proposed to predict antioxidant proteins, and the accuracy of the method is 66.88% in jackknife test [8]. AodPred [9] is a method based on SVM classifier by using g-gap dipeptide features. The accuracy of AodPred based on g-gap dipeptides is 74.79% in jackknife test. Thus, the experimental results demonstrate that our method can attain high accuracy and classify antioxidant and non-antioxidant proteins efficiently. The time complexity of computation method depends on the classification method SVM, which is related to the number of training samples and the feature dimension.

### 2.2. The Comparison of Performance Evaluation on Feature Selection Methods

**Figure 1:**
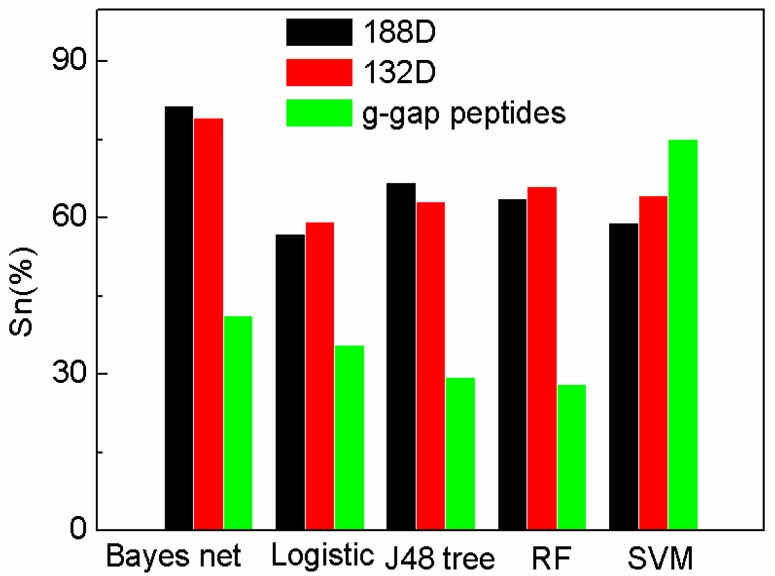
Comparison of our features with g-gap using different classifiers on Sn.

**Figure 2:**
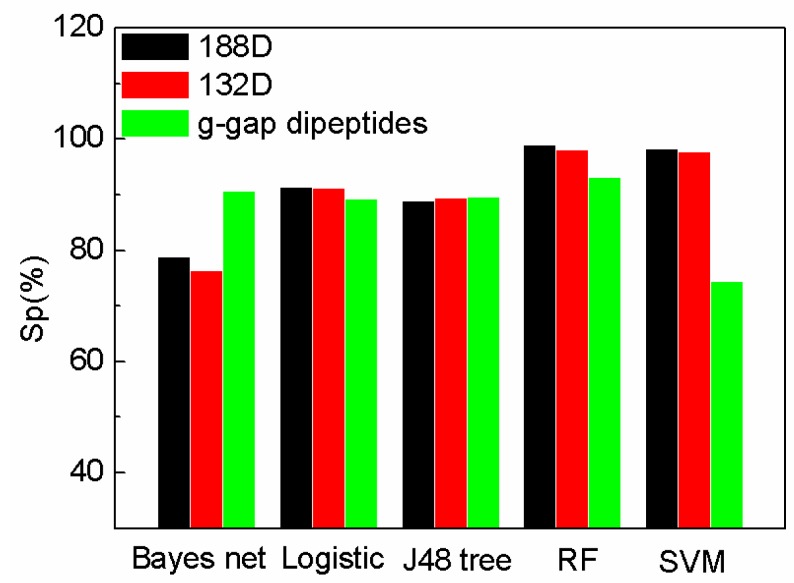
Comparison of our features with g-gap using different classifiers on Sp.

**Figure 3:**
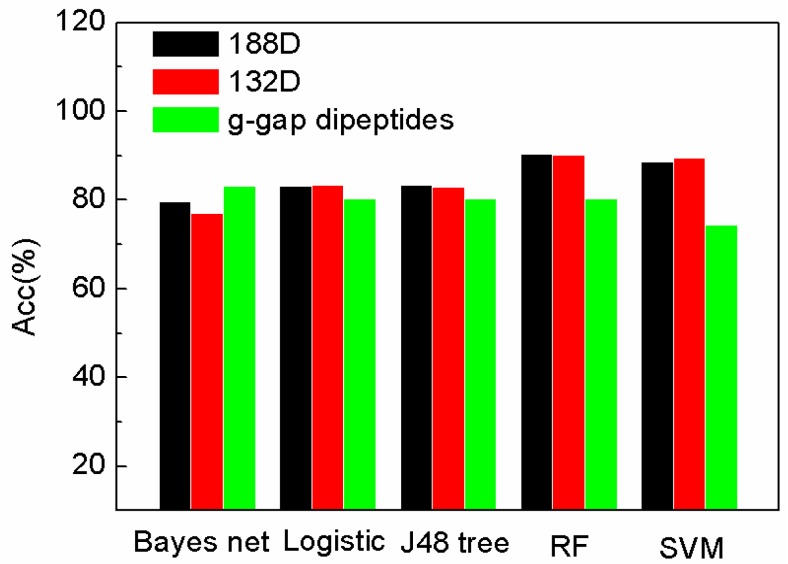
Comparison of our features with g-gap using different classifiers on Acc.

To further demonstrate the performance of our sequence-based method and the selected 132D features, the features are compared with g-gap dipeptides by using other classifiers provided by WEKA [29]. The feature set of 188D is reduced by MRMD method to 132D. MRMD method is a feature method, which is mentioned in Section 3.6. The performance of the features on different classifiers on sensitivity (Sn), specificity (Sp), and accuracy (Acc) are compared in Figure 1, Figure 2 and Figure 3. In Figure 1, Figure 2 and Figure 3, “Logistic” is short for logistic regression. J48 tree is a decision tree method based on C4.5. RF and SVM are short for random forest and support vector machine. 

The Sn on 132D used Bayes net performs better than other methods. In the experiments, we can see that our method (188D and 132D using SVM) performs better than other classifiers using g-gap dipeptides, except SVM. However, Bayes net using 188D attains the highest Sn with 81.6%. The Sn of reduced 132D on Bayes net also performs better than that of AodPred. Figure 1 also shows that 188D and 132D perform better than g-gap dipeptides on most classifiers, which means that 188D and 132D are more robust than g-gap dipeptides. The figure also shows that the reduced 132D removes the redundancy, and can attain comparably high sensitivity on Bayes net and J48 tree. The sensitivity of 132D reduced features is higher than that of 188D on the other three classifiers. Thus, it is necessary to select features by max relevance max distance method.

The comparison of specificity with the features on different classifiers is shown in Figure 2. Our method (188D with SVM) performs better than that of AodPred (g-gap dipeptides) on specificity. The value of Sp of the reduced SeqSVM is higher than that of AodPred (g-gap dipeptides). G-gap dipeptides performs on Bayes net than 188D and 132D. The values of Sp using different features are comparable on Logistic, J48 tree, and RF classifiers.

In Figure 3, the accuracy of SeqSVM with 188D and 132D is better than that of AodPred (g-gap dipeptides SVM).

### 2.3. The Comparison of SeqSVM

The method of SeqSVM with SMOTE is compared to SeqSVM without SMOTE. The comparison of SeqSVM methods is shown in Table 2. The accuracy of SeqSVM before SMOTE is 85.98%, while the accuracy of SeqSVM is 88.68% after SMOTE processing. The accuracy of SeqSVM is improved by 3.1% after using SMOTE processing compared with SeqSVM without SMOTE processing. The accuracy of SeqSVM with SMOTE and MRMD is 89.46%, which the accuracy is improved by 4% compared with SeqSVM. The experimental results demonstrate that the performance of classifier can be improved by using SMOTE processing, when the number of class sample is imbalance. Although the computational methods can attain an encouraging classification result, the experimental results are verified based on the biochemical approaches, such as wet biochemistry and molecular biology techniques.

## 3. Materials and Methods

### 3.1. Benchmark Dataset

The dataset used in our work is generated and used by Feng et al. [8,30,31], and the data are selected from the UniProt database. For the purpose of selecting valid data, only the proteins that have been confirmed with antioxidative activities are selected, and the proteins with ambiguous meanings (such as “B”, “X”, “Z”) are excluded. The benchmark dataset (S) is represented by positive subset (S^+^) and negative subset (S^−^), formulated as Equation (1).

(1) $$S=S^{+}\cup S^{-},$$

where the symbol “∪” means the union in the set theory. There are 710 antioxidant proteins and 1567 non-antioxidant proteins left after the selection process. Furthermore, the selected sequences contain redundancy with high similarity. To avoid the overestimation of the methods, the homologous sequences with more than 60% similarity are removed by CD-HIT program [32] from the dataset. Finally, a benchmark dataset with 253 antioxidant proteins and 1552 non-antioxidant proteins is used for the prediction model. As a result, the positive subset (S^+^) contains 253 samples, while there are 1552 samples in the negative subset (S^−^).

### 3.2. Support Vector Machine

Support vector machine (SVM) is a supervised classification model. As we have known, SVM has been widely used in bioinformatics [9,33,34,35,36,37,38,39,40,41,42,43,44,45,46], so here, we introduce it briefly. In linearly separable cases, the key idea of SVM is that a hyperplane is built to separate the two groups with a maximum margin. If the samples are non-linearly separated, the input variables are mapped into a high dimensional feature space by a kernel function. The principle of SVM is introduced in [47,48], and more details are provided in [49]. The SVM used in our work is the package named LIBSVM written by Chang and Lin [50]. Radial kernel function (RBF) is selected because of its effectiveness and efficiency. The regularization parameter, C, and the kernel width parameter, γ, are optimized by the grid search approach.

### 3.3. SMOTE Processing

There are 253 antioxidant proteins and 1552 non-antioxidant proteins in the dataset. The dataset is quite imbalanced for the reason that the positive samples and negative samples are not equally represented. SMOTE [51] is an approach to achieve a better result by oversampling the minority class and undersampling the majority class. The key idea of SMOTE is that a synthetic sample is created by oversampling method, instead of replacement. The minority class is composed of the minority class samples and the synthetic samples. The synthetic samples are generated along the line segments joining any or all of the K minority class nearest neighbors. If 200% samples should be oversampled, two out of K nearest samples will be chosen, and samples are generated on each direction of the chosen neighbors. The data are standardized after SMOTE processing.

### 3.4. Sequence Representation

**Figure 4:**
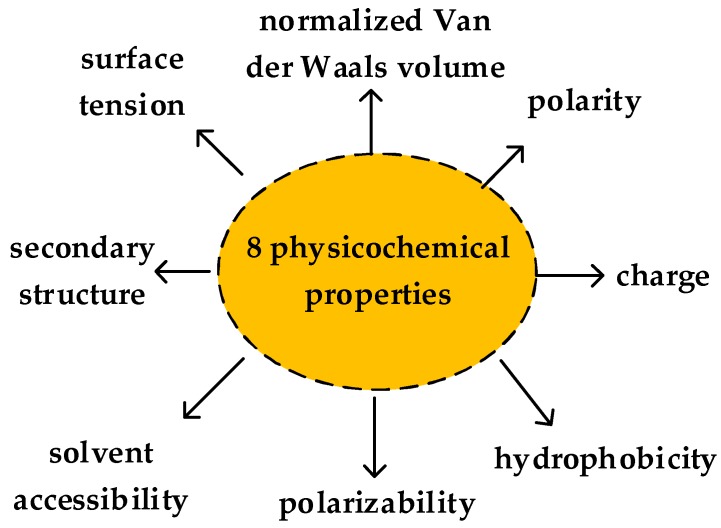
Eight physicochemical property attributes.

188D vector was used to extract features of proteins by Cai et al. in 2003 [17]. The property of 188D includes the amino acid composition, distribution and physicochemical property. Due to the diversity of the amino acid, to extract the features clearly, the mentioned properties are divided into four classes. C1 means the percentage of amino acid (based on the amino acid class), C2 represents the percentage of amino acid (based on physicochemical property). There are 20 amino acids, so the dimension number of frequency of each amino acid is 20. The physicochemical property is represented by eight attributes, which are secondary structure, solvent accessibility, normalized Van der Waals volume, hydrophobicity, charge, polarity, polarizability, and surface tension. There are three values for each attribute, for example, the attribute of secondary structure can be described by EALMQKRH, VIYCWFT, or GNPSD, denoted by R_ij_ (1≤i≤8, 1≤j≤3). The physicochemical property of proteins is shown as Figure 4. Thus, 24 attributes are used for describing the physicochemical properties. B describes the percent frequency of bivalent. There are three types of bivalent used for each property, denoted by $R_{\mathrm{im}}R_{\mathrm{in}},R_{\mathrm{im}}R_{\mathrm{io}},R_{\mathrm{in}}R_{\mathrm{io}}\left(1\leq m,n,o\leq 3\right)$. Thus, there are 24 dimensions on the eight physicochemical property attributes.

Given a protein sequence with length L, the percent of the amino acids of a particular property located at the first, 25%, 50%, 75%, 100% is measured as the distribution of the protein. There are 24 attributes used to describe the physicochemical properties. The distributions of amino acids are represented by 120 attributes, by the reason that there are five values on each attribute. Above all, the total number of attributes for protein representation is 188. In fact, it is obvious that not all of the 188 features will be used for prediction. There is redundancy between the features. Thus, the features are selected by max relevance max distance method proposed by Zou [28].

### 3.5. Performance Evaluation

Sensitivity (Sn), specificity (Sp), and accuracy (Acc) are used to measure the classification quality. Sensitivity is used in Chou’s work [52,53,54,55], and represents the sensitivity, which is calculated by Equation (2). Specificity is the specificity of the algorithm, which is measured by the rate of misclassification of the antioxidant proteins. The calculation of Sp is shown as Equation (3). Assessments of Sp or Sn, individually, are not sufficient to evaluate the performance of a method. The overall accuracy is calculated by Equation (4).

(2) $$S_{n=\frac{\mathrm{TP}}{\mathrm{TP}+\mathrm{FN}}},$$

(3) $$S_{p=\frac{\mathrm{TN}}{\mathrm{TN}+\mathrm{FP}}},$$

(4) $$\mathrm{Acc}=\frac{\mathrm{TP}+\mathrm{TN}}{\mathrm{TP}+\mathrm{FN}+\mathrm{TN}+\mathrm{FP}},$$

where TP is the number of true positive samples, TN represents the number of true negative samples, FN represents the number of false negative samples, and FP represents the number of false positive samples. 

Assume N^+^ is the number of antioxidant proteins labeled by the classification method, and N^−^ is the number of non-antioxidant proteins labeled by the classification method. $N_{-}^{+}$ is the number of antioxidant proteins which are misclassified by non-antioxidant proteins. $N_{+}^{-}$ is the number of non-antioxidant proteins which are mislabeled by antioxidant proteins. Thus, there are

(5) $$\mathrm{TP}=N^{+}-N_{-}^{+},$$

(6) $$\mathrm{TN}=N^{-}-N_{+}^{-},$$

(7) $$\mathrm{FP}=N_{+}^{-},$$

(8) $$\mathrm{FN}=N_{-}^{+}.$$

If $N_{-}^{+}=0$, this means that all antioxidant proteins are recognized, and the sensitivity Sn = 1. Similarly, if $N_{+}^{-}=0$, this means that none of the non-antioxidant proteins are misclassified as antioxidant proteins, and the value of specificity Sp = 1. Equations (9)–(11) can be rewritten as

(9) $$\mathrm{Sn}=1-\frac{N_{-}^{+}}{N^{+}},$$

(10) $$\mathrm{Sp}=1-\frac{N_{+}^{-}}{N^{-}},$$

(11) $$\mathrm{Acc}=1-\frac{N_{-}^{+}+N_{+}^{-}}{N^{-}+N^{+}}.$$

From Equations (9)–(11), it is obvious that if $N_{+}^{-}=N_{-}^{+}=0$, which means that none of the antioxidant peptides or the non-antioxidant peptides are misclassified. Thus, there is Sn = Sp = Acc = 1. The values of Sn, Sp, and Acc are larger, and the performance of the method is better. 

In the experiments, the predictors are evaluated by the jackknife cross-validation [56]. There are three cross-validation test methods used in the literature, which are independent dataset test, K-fold cross-validation (i.e., 5-fold cross-validation or 10-fold cross-validation) and jackknife cross-validation test [56]. Jackknife test is considered as the least arbitrary and most objective [57]. The advantage of jackknife test has been demonstrated in that it can give a unique output for a given benchmark dataset. 

### 3.6. Feature Selection

Feature selection techniques have been widely applied to problems in bioinformatics [57,58,59,60,61]. In this work, we use maximum relevance maximum distance (MRMD) [28] to remove the redundancy of features. The objective function of MRMD is shown as Equation (12). If m^−1^ features have been selected, the *m*-th feature will be selected if the *i*-th feature maximizes Equation (12).

(12) $$\max\left(MR_{i}+MD_{i}\right)$$

where *MR_i_* is the relevance between the features. The relevance is measured by the Pearson’s correlation coefficient, shown as Equation (13).

(13) $$PCC\left(\vec{X},\vec{Y}\right)=\frac{\sum_{k=1}^{N}\left(x_{k}-\bar{x}\right)\left(y_{k}-\bar{y}\right)}{\sqrt{\sum_{k=1}^{N}\left(x_{k}-\bar{x}\right)}\sqrt{\sum_{k=1}^{N}\left(y_{k}-\bar{y}\right)}},$$

where *N* is the number of vectors, and $\bar{x}\left(\bar{y}\right)$ is the average value on the *k*-th dimension. *MD_i_* is used to measure the level of similarity between two feature vectors. In our experiments, the maximum distance is calculated as the mean of the Euclidean distance (*ED*), cosine distance (*COS*), and Tanimoto coefficient (*TC*) (shown as Equation (16)). The distances used are defined as follows.

(14) $$ED_{i}=\frac{\sum ED\left({\vec{F}}_{i},{\vec{F}}_{k}\right)}{M-1}=\frac{\sum \sqrt{\sum_{k=1}^{K}{\left(x_{i}-x_{k}\right)}^{2}}}{M-1}\;\;\left(1\leq k\leq M,k\neq i\right),$$

(15) $$COS_{i}=\frac{\sum cos\left({\vec{F}}_{i},{\vec{F}}_{k}\right)}{M-1}=\frac{\sum {\vec{F}}_{i}{\vec{F}}_{k}/\left|\left|{\vec{F}}_{i}\right|\right|\left|\left|{\vec{F}}_{k}\right|\right|}{M-1}\;\;\left(1\leq k\leq M,k\neq i\right),$$

(16) $$TC_{i}=\frac{\sum TC\left({\vec{F}}_{i},{\vec{F}}_{k}\right)}{M-1}=\frac{\sum {\vec{F}}_{i}{\vec{F}}_{k}/({\left|\left|{\vec{F}}_{i}\right|\right|}^{2}+{\left|\left|{\vec{F}}_{k}\right|\right|}^{2}-{\vec{F}}_{i}{\vec{F}}_{k})}{M-1}\;\;\left(1\leq k\leq M,k\neq i\right),$$

(17) $$maxMD_{i}=\frac{1}{3}\left(ED_{i}+COS_{i}+TC_{i}\right)\;\;\left(1\leq i\leq M\right),$$

where *M* is the number of features. The distance is calculated on each dimension, and the feature will be selected with the maximum distance by satisfying the condition of Equation (17). 

## 4. Conclusions

Antioxidant proteins can terminate the cellular and DNA damage caused by external sources, such as exposures to X-rays, ozone, cigarette smoking, and others. The study of antioxidant proteins has drawn attention in recent years. The computational methods have been proposed to identify the antioxidant proteins, and the results are encouraging. In our work, a method based on primary sequence information, using SVM, is proposed to predict antioxidant proteins, and the experimental results show that our method performs better than existing methods. The contribution of our work is that a computational method is proposed to predict antioxidant proteins, and the classification accuracy of the method is better than some existing methods. Since there are publicly accessible web servers provided for practical models [62,63,64,65,66], the web server for identifying antioxidant proteins based on our method will be developed later to help the researchers identify the antioxidant proteins. We will also extend our work to other organism in our future work, such as *E. coli*/*S. cerevisiae*/*D. radiodurans* in UniProt database.

## Figures and Tables

**Table 1:** The comparison of accuracy with existing methods.

Performance Evaluation	SeqSVM (132D)	AodPred	Nave Bayes
Accuracy	89.46%	74.49%	66.88%

**Table 2:** The comparison of accuracy on SeqSVM methods.

Performance Evaluation	SeqSVM (Non-SMOTE)	SeqSVM (SMOTE)	SeqSVM (SMOTE + MRMD)
Accuracy	85.98%	88.68%	89.46%
